# Monoamine Oxidase A Gene (MAOA) Associated with Attitude Towards Longshot Risks

**DOI:** 10.1371/journal.pone.0008516

**Published:** 2009-12-31

**Authors:** Songfa Zhong, Salomon Israel, Hong Xue, Richard P. Ebstein, Soo Hong Chew

**Affiliations:** 1 Center for Experimental Business Research and Department of Economics, Hong Kong University of Science and Technology, Kowloon, Hong Kong Special Administrative Region; 2 Scheinfeld Center of Human Genetics for Social Sciences, Hebrew University, Jerusalem, Israel; 3 Applied Genomics Laboratory and Department of Biochemistry, Hong Kong University of Science and Technology, Hong Kong Special Administrative Region; 4 Herzog Memorial Hospital, Givat Shaul, Jerusalem, Israel; 5 Department of Economics and Department of Finance, National University of Singapore, Singapore, Singapore; California Institute of Technology, United States of America

## Abstract

Decision making often entails longshot risks involving a small chance of receiving a substantial outcome. People tend to be risk preferring (averse) when facing longshot risks involving significant gains (losses). This differentiation towards longshot risks underpins the markets for lottery as well as for insurance. Both lottery and insurance have emerged since ancient times and continue to play a useful role in the modern economy. In this study, we observe subjects' incentivized choices in a controlled laboratory setting, and investigate their association with a widely studied, promoter-region repeat functional polymorphism in monoamine oxidase A gene (*MAOA*). We find that subjects with the high activity (4-repeat) allele are characterized by a preference for the longshot lottery and also less insurance purchasing than subjects with the low activity (3-repeat) allele. This is the first result to link attitude towards longshot risks to a specific gene. It complements recent findings on the neurobiological basis of economic risk taking.

## Introduction

In one form or another, longshot risks touch the lives of everyone. On the gain side, we see a wide array of lottery products beyond the traditional state lotteries and the emergence of lottery consortia such as Powerball and Mega Millions. Sweepstakes or lucky draws are often bundled with the purchase of goods such as magazines, credit cards, automobiles, even homes. Because lotteries are invariably priced higher than their expected payoffs, they are used to raise revenues for charities, clubs, organizations, and governments. In economics parlance, we say that lottery consumers are risk seeking when they pay more than the expected payoffs in purchasing lottery products. Moreover, in lottery markets, e.g., racetrack betting, there is a tendency for consumers to exhibit a preference for longshot – valuing longshot bets with higher odds more than favorites with lower odds when expected payoffs are similar [1], [2].

On the loss side, we observe that much of the hazards in life, including accident, fire, and illness, be covered by insurance. An ancient example of insurance was practiced by Babylonian traders as far back as 2 millennia BC, as recorded in the Code of Hammurabi. If a Mediterranean sailing merchant received a loan to fund his shipment, an additional sum could be paid to the lender in exchange for the guarantee to cancel the loan, should the shipment be stolen. Insurance customers are risk averse in paying more than the expected loss to shift their liability for loss to the insurance company.

Why do people concurrently buy lottery and insurance? In an early attempt to address this puzzle within the framework of the expected utility model [3], [4]. Friedman and Savage [5] proposed a reversed S shaped utility function which is concave for a large range of outcome values and convex for extremely high gains. Dissatisfaction with the lack of empirical validity of the expected utility model [6], [7] led to the development of non-expected utility models including prospect theory [8], [9]. Besides a utility function that is concave over gains and concave over losses, they introduce a probability weighting function which generally underweights probabilities except for small probabilities when it overweights and show that prospect theory can account for the concurrence of lottery and insurance purchase for the same economic agent.

Recently, the heritability of economic risk taking has been investigated using twin studies [10], [11]. At the same time, identification of specific genes associated with risk taking has been investigated in several recent studies [12]–[15]. Yet, the neurogenetic correlates of attitudes towards longshot risks, such as lottery and insurance purchase, remain unexplored.

Monoamine oxidase A, *MAOA* (MIM 309850), contains 15 exons and is located on chromosome Xp11.23 [16]. During embryonic development *MAOA* is the main catabolic enzyme for degradation of both dopamine and norepinephrine [17] suggesting a possible impact on adult behavior. In *MAOA* knockout pup brains, serotonin concentrations were increased up to ninefold, and serotonin-like immunoreactivity was present in catecholaminergic neurons. In pup and adult brains, norepinephrine concentrations were increased up to twofold, and cytoarchitectural changes were observed in the somatosensory cortex [18]. Sabol et al. [19] described an upstream polymorphism located 1.2 kb from the coding sequences that consists of a 30-bp repeat sequence present in 3, 3.5, 4, or 5 copies. Importantly, the polymorphism impacts on the transcriptional efficiency of the gene and repeats with 3.5 or 4 copies of the repeat sequence are transcribed 2–10 times more efficiently than those with 3 or 5 copies of the repeat. In most populations the 3 and 4 repeats are most common, including Chinese.

There have been several studies linking MAOA u-VNTR on brain function during cognition, emotional arousal and personality tests [20]–[23]. In particular, Meyer-Lindenberg et al. [22] showed that the low-expression variant of MAOA (3-repeat allele) predicted hyperresponsive amygdala during emotional arousal, with diminished reactivity of regulatory prefrontal regions, compared with the high-expression allele (4-repeat allele), suggesting that dysregulated and hyperreactive amygdala response may contribute to increased anxiety. Buckholz et al. [23] demonstrated that male carriers of the low-expressing genetic variant (3-repeat allele) exhibited dysregulated amygdala activation and increased functional coupling with ventromedial prefrontal cortex (vmPFC). Stronger coupling predicted increased Tridimensional Personality Questionnaire (TPQ) harm avoidance [24] and decreased reward dependence scores, suggesting that this circuitry mediates a part of the association of MAOA with these traits. These results prompted us to examine the role of the MAOA promoter-region repeat in partially explaining individual differences in decision making under risk. We hypothesized that individuals with the MAOA high activity allele, which is more responsive to emotional arousal based on the Meyer-Lindenberg et al. [22] observation and mediates amygdala-vmPFC coupling and harm avoidance based on the Buckholz et al. [23] observation, would show a preference for longshot and be less disposed to purchasing insurance.

## Methods

### Ethics Statement

Each subject gave informed written consent for participation both in the economic experiment and in having his/her blood sample taken. The study including the use of subject payment incentive in the economic experiment and collection of blood sample was approved by the Institutional Review Board at the Hong Kong University of Science and Technology.

### Subjects

We recruited a cohort of 350 Han Chinese subjects in Beijing through internet advertisement, posters and word of mouth to assess their risk attitude and genotype the *MAOA* polymorphism. The first group was recruited in July 2007; the second group was recruited in February 2008. Demographics of the subjects are summarized as follows: mean age 28.2 +/− 10.8; 162 male, 188 female; 123 non-student subjects, 227 student subjects; 67 subjects with high school education, 194 subjects with college education, 89 subjects with postgraduate education; 325 Han Chinese, 25 non-Han Chinese. Only the 325 Han Chinese are included in the analysis. We adhere to the practice in experimental economics of applying monetary incentive to motivate decision making without using deception. After the experiment, subjects each donated 10 cc of blood for genotyping.

### Economic Experiment

We use two simple choice tasks to represent lottery and insurance. In the first task which concerns the lottery, subjects are tasked to rank three items: (A) 1% chance of receiving Y200 and zero otherwise, (B) 10% chance of receiving Y20 and zero otherwise, (C) receiving Y2 for sure. We paid subjects their most preferable choice as incentive. Subjects are classified as exhibiting longshot preference, when A is preferred to B which is in turn preferred to receiving C. In the second task concerning insurance, subjects are classified as being disposed to insure if they prefer losing Y2 for sure than losing Y2000 with 0.1% chance. Given the size of the loss involved, we did not incentivize the insurance task.

### Genotyping

The Polymorphism for the 3- and 4 promoter region repeats of MAOA was characterized using PCR amplification procedure with the following primer: F5′- ACAGCCTGACCGTGGA-3′, R5′- GAACGGACGCTCCATT-3′. PCR reactions were performed using 5 µl Master Mix (Thermo scientific), 2 µl primers (0.5 µM), 0.6 µl Mg/Cl2 (2.5 mM), 0.4 µl DMSO 5% and 1 µl of water to total of 9 µl total volume and an additional 1 µl of genomic DNA was added to the mixture. All PCR reactions were employed on a Biometra T1 Thermocycler (Biometra, Güttingem, Germany). PCR reaction condition was as follows: preheating step at 95.0°C for 5 min, 29 cycles of denaturation at 95.0°C for 40 s, reannealing at 64°C for 40 s and extension at 72°C for 60 s. The reaction proceeded to a hold at 72°C for 5 min. The mixtures were electrophoresed on a 4% agarose gel (AMRESCO) with ethidium bromide to screen for genotype. The allele and genotype frequency is summarized in Table 1.

**Table 1:** Distribution of Allele and genotype frequency of MAOA gene.

	Allele			Genotype	
3	4	5	3/3	3/4	4/4
59.7%	40.6%	0.5%	46.8%	24.1%	28.5%

### Statistical Analysis

Since our dependent variable, longshot preference/insurance purchase, is binary, we use logit regression with genes, age, sex, student status and education as independent variables with robust standard error for both genotype and allele association analysis. We also use bivariate probit regression model with genes, age, sex, student status and education as independent variables with robust standard error for both genotype and allele association analysis, to estimate jointly the effect of MAOA on lottery and insurance purchase. For the allele model, given that one data point for each allele gives rise to two data points for each subject, we used subject ID as a cluster variable for robust standard errors. For the genotype model, we report results of the additive model which assumes the absence of dominant genetic effects of any particular allele.

## Results

As previously observed, 31.2% of the subjects exhibit longshot preference and 26.1% of the subjects purchase the insurance. Male subjects exhibit longshot preference significantly more frequently thawen female subjects (p = 0.010), but not insurance purchase (p = 0.209). This is consistent with previous studies by Fehr-Duda et al. [25] and Booij et al. [26], both of which showed that males less risk averse than females in the gain domain but not in the loss domain. Older subjects significantly tend to purchase insurance more than the younger subjects (p = 0.001), but not longshot preference (p = 0.315).

**Figure 1:**
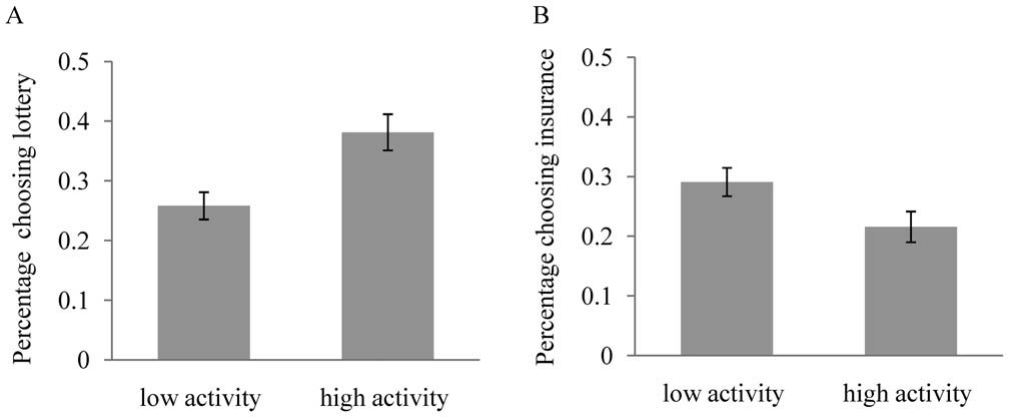
MAOA gene, lottery, and insurance. (A). MAOA and lottery. Subjects with the high activity allele (4-repeat allele) are significantly more likely to exhibit longshot preference than subject with the low activity allele (3-repeat allele). (B). MAOA and insurance. Subjects with the low activity allele (3-repeat allele) are more likely to exhibit preference for insurance than subject with the high activity allele (4-repeat allele).

We test the effect of MAOA on lottery and insurance purchase with four controls – age, gender, student status and education – and find that it is significantly associated with longshot preference (allele model, p = 0.006; genotype model, p = 0.007), and marginally associated with insurance purchasing (allele model, p = 0.086 genotype model, p = 0.085). Subjects with the 4-repeat allele (high activity) of *MAOA* are characterized by a preference for the lottery and less insurance purchase than subjects with 3-repeat allele (low activity). In separate gender analysis, the association between *MAOA* and longshot preference remains significant. The relevant statistics are summarized in Figure 1 and Table 2.

**Table 2:** Association of lottery and insurance purchase with MAOA.

Longshot Risk	Subject	Allele Model				Genotype Model			
		*OR*	*CI*		*p-value*	*OR*	*CI*		*p-value*
Lottery	Pooled	0.548	0.356	0.842	0.006	0.674	0.506	0.897	0.007
*(High activity*	Male	0.509	0.247	1.048	0.067	0.714	0.497	1.024	0.067
*allele)*	Female	0.597	0.365	0.975	0.039	0.619	0.388	0.988	0.044
Insurance	Pooled	1.511	0.944	2.422	0.086	1.323	0.962	1.820	0.085
*(Low activity*	Male	1.753	0.753	4.081	0.193	1.324	0.868	2.020	0.193
*Allele)*	Female	1.335	0.779	2.287	0.292	1.301	0.799	2.118	0.290

Logit regression is used to test the effect of MAOA on lottery and insurance purchase with control variables of age, gender, student status, and education. This table reports results using both allele and genotype models for pooled, male, and female subjects. OR refers to odds ratio and CI refers to its 95% confidence interval.

Since each individual makes both lottery and insurance purchase decisions, their residuals may be correlated with each other. We apply bivariate probit regression to address this unobserved effect and find that the effect of MAOA remains significant for lottery purchase (allele model, p = 0.010, genotype model, p = 0.011) and marginally significant for insurance purchase (allele model, p = 0.088, genotype model, p = 0.088). We also find that the residuals for lottery and insurance purchases are not significantly correlated (p-value for Lagrangian multiplier statistic = 0.467). This supports the appropriateness of our choice of the cross section logit regression model (Table 2).

## Discussion

Several recent papers have explored the molecular genetic basis of economic risk taking. With 95 subjects, Dreber et al. [12] showed that the dopamine receptor D4 gene (DRD4) exon 3 repeats are associated with financial risk taking. This was replicated independently in a 65-subject study by Kuhnen & Chiao [13] who found additionally an association with the serotonin transporter (5-HTTLPR). Zhong et al. [15] proposed a neurochemical model relating dopamine and serotonin tones respectively to valuation sensitivity over gains and losses and derived its implication on risk attitude over risks involving moderate probabilities. They tested and validated their hypothesis with a gene association experiment showing that dopamine transporter (DAT1) is associated with risk attitude over gains and that an intronic 17 bp variable number of tandem repeat of serotonin transporter (STin2) is associated with risk attitude over losses. Roe et al. [14] showed that economic risk attitude is associated with several vesicular monoamine transporter (VMAT2) SNPs. The present paper is the first investigation of the neurogenetic correlates of attitude towards longshot risks observed through laboratory-based economic experiments. Our findings complement existing evidence about the role of MAOA in the modulation of personality traits including harm avoidance [23].

The neurogenetics strategy is a powerful tool that enables researchers to identify the neurochemical pathways encoded by genetic variations underpinning individual differences in human decision making. This approach adds to the emerging literature on the neuroeconomics of decision making (see, e.g., [27], [28]), especially previous studies on decision making under risk [29], [30]. As observed in Benjamin et al., [31], introducing a molecular genetics approach would potentially enhance the predictive power of economic theory. With more empirical validation of our neurogenetic results suggesting that individual differences in preference over longshot risks are partially hard-wired traits, the stage is set for testing their implication in institutional and market settings.
